# Mental Health in the Digital Age: Grave Dangers, Great Promise

**DOI:** 10.1192/pb.bp.116.054650

**Published:** 2017-06

**Authors:** Melvyn W. B. Zhang

Technology has always been a double-edged sword: there are associated risks and benefits. As a practising psychiatrist I increasingly rely on technology at work, using next-generation electronic medical records and at times recommending appropriate smartphone-based applications as additional therapy for my patients.

In contrast to numerous other titles about technology and its impact on healthcare – which have emerged as a result of the massive technical advances in the past decade – *Mental Health in the Digital Age* does not focus only on the benefits of the use of technology in mental healthcare. It offers a timely balanced perspective by also providing an in-depth analysis of the risks.

The risks highlighted in the book are not limited to addictive behaviours such as internet or gaming addiction, but also include cyberbullying and the increased risk of suicide due to pro-suicide websites and suicide pacts. Cyberbullying is perhaps one of the most common problems linked with the use of technology to date and it is not unusual for me and my team to see children and adolescents who refuse to go to school as a result of cyberbullying. Unlike conventional forms of bullying, cyberbullying implies the use of social networks and internet-based messaging services to harass an individual. This work examines not only the prevalence of the problem, but also the various prevention strategies available, such as having a specific academic curriculum to deal with the issue.

The authors review the existing literature comprehensively – referring also to current evidence – and look at the potential of technology across several areas of mental healthcare, including the provision of psychotherapy and the integration of patients' health records. They also discuss how recent advances – such as virtual reality – could in principle be a powerful tool in exposure therapy. As a team with an interest in e-health, my colleagues and I have been developing smartphone applications for various mental health disorders. The introduction of virtual reality technology means that we could perhaps tap on games and various other sensors and headset devices to create an interactive environment not just for psychotherapy but for other forms of interventions too.

This is a good guide for novices in e-health but equally a useful tool for the more experienced in this area. It would be helpful if a future edition included more detailed coverage of smartphone applications and their inherent risks and benefits – a topic of concern not only for clinicians, but patients at large.

